# Biostimulation of indigenous microbes for uranium bioremediation in former U mine water: multidisciplinary approach assessment

**DOI:** 10.1007/s11356-023-31530-4

**Published:** 2023-12-29

**Authors:** Antonio M. Newman-Portela, Evelyn Krawczyk-Bärsch, Margarita Lopez-Fernandez, Frank Bok, Andrea Kassahun, Björn Drobot, Robin Steudtner, Thorsten Stumpf, Johannes Raff, Mohamed L. Merroun

**Affiliations:** 1 Department of Microbiology, Faculty of Science, University of Granada, Avda. Fuentenueva S/N, 18071 Granada, Spain; 2 Institute of Resource Ecology, Helmholtz-Zentrum Dresden-Rossendorf, Bautzner Landstraße 400, 01328 Dresden, Germany; 3 WISMUT GmbH, Jagdschänkenstraße 29, 09117 Chemnitz, Germany

**Keywords:** Mine water, Uranium, Bacterial communities, Fungal communities, Bioremediation, Electron donors, Glycerol, Bioreduction

## Abstract

Characterizing uranium (U) mine water is necessary to understand and design an effective bioremediation strategy. In this study, water samples from two former U-mines in East Germany were analysed. The U and sulphate (SO_4_^2−^) concentrations of Schlema-Alberoda mine water (U: 1 mg/L; SO_4_^2−^: 335 mg/L) were 2 and 3 order of magnitude higher than those of the Pöhla sample (U: 0.01 mg/L; SO_4_^2−^: 0.5 mg/L). U and SO_4_^2−^ seemed to influence the microbial diversity of the two water samples. Microbial diversity analysis identified U(VI)-reducing bacteria (e.g. *Desulfurivibrio*) and wood-degrading fungi (e.g. *Cadophora*) providing as electron donors for the growth of U-reducers. U-bioreduction experiments were performed to screen electron donors (glycerol, vanillic acid, and gluconic acid) for Schlema-Alberoda U-mine water bioremediation purpose. Thermodynamic speciation calculations show that under experimental conditions, U(VI) is not coordinated to the amended electron donors. Glycerol was the best-studied electron donor as it effectively removed 99% of soluble U, 95% of Fe, and 58% of SO_4_^2−^ from the mine water, probably by biostimulation of indigenous microbes. Vanillic acid removed 90% of U, and no U removal occurred using gluconic acid.

**Supplementary Information:**

The online version contains supplementary material available at 10.1007/s11356-023-31530-4.

## Introduction

Uranium (U) mining and processing have their origins in the second half of the twentieth century in East Germany, mainly in the Federal States of Saxony and Thuringia (Bernhard et al. 1998; Albrecht 2017). Intense mining activities are a major source of soluble U, which can migrate into surrounding aquifers, representing a significant environmental and human health threat (Jroundi et al. 2020; Lopez-Fernandez et al. 2021). It is well known that U toxicity depends upon its chemical speciation, which is in turn controlled by abiotic and biotic processes. Therefore, understanding the U speciation in mine water from mines is essential to predict possible U migration in the environment and to design efficient remediation technologies (Newsome et al. 2014).

Conventional remediation strategies have focused on physical and chemical processes such as controlled flooding of mine galleries, permeable reactive multi-barriers, chemical precipitation, and solvent extraction. However, these approaches are time consuming, economically infeasible, and not very effective for very low U concentrations (Sánchez-Castro et al. 2021; Banala et al. 2021). These remediation technologies should meet the set water quality regulatory standard for beneficial reuse of the U mine water for different purposes (e.g. irrigation, especially in water-stressed regions in the world) within the concept of circular economy (Annandale et al. 2017). Bioremediation of U based on the interaction of biological agents (e.g. plants, algae, fungi, and bacteria) with this radionuclide could be considered as an innovative and promising alternative (Kalin et al. 2005; Gadd and Fomina 2011; Chen et al. 2021; You et al. 2021). Micro-organisms can interact with U through mechanisms such as biomineralization, enzymatic reduction, biosorption, and intracellular accumulation, altering its speciation and playing an important role in the solubility and mobility of this radionuclide in aquatic environments (Merroun and Selenska-Pobell 2008; Gallois et al. 2018; Lopez-Fernandez et al. 2021; You et al. 2021). U bioremediation strategies are mainly based on U phosphate biomineralization under aerobic conditions (Jroundi et al. 2007; Krawczyk-Bärsch et al. 2015; Sánchez-Castro et al. 2020; Martínez-Rodríguez et al. 2023), and bioreduction under anaerobic conditions (Lovley et al. 1991; Phillips et al. 1995; Newsome et al. 2014).

Biological enzymatic reduction of U(VI) to U(IV) has been the objective of several research studies over recent decades in U-contaminated groundwater masses (Lovley et al. 1991; You et al. 2021). In terrestrial environments, U usually occurs in either the hexavalent or tetravalent oxidation state. Hexavalent U (U(VI)) is soluble, mobile and therefore bioavailable in oxic conditions. However, tetravalent U (U(IV)) is insoluble and immobile, substantially decreasing its bioavailability along with its potential toxicity (Krawczyk-Bärsch et al. 2018; Lopez-Fernandez et al. 2021). The immobilization of highly soluble U(VI) (e.g. UO_2_^2+^) to the insoluble U(IV) mineral, such as uraninite (UO_2_), occurs through bacterial reduction under anoxic conditions. This transition could occur as a direct process, where U(VI) acts as a final electron acceptor, or as an indirect process, coupled to the microbial reduction of Fe(III) (Liu et al. 2007; You et al. 2021). In a natural environment, this reduction process is not carried out by a single micro-organism, but rather a microbial consortium including U reducing bacteria, generating optimal conditions for biological reduction to take place. For instance, fungi are widely distributed in former mines, which have been treated by controlled flooding (Arnold et al. 2011; Kassahun et al. 2018) and are considered as a source of electron donors needed for the U bioreduction. Heterotrophic microbes (e.g. fungi) in a mine gallery degrade wood and consume oxygen, producing organic compounds such as saccharic acids, glycerol, and vanillin. These compounds can serve as electron donors for U reducing bacteria (Baraniak et al. 2002; Haq et al. 2022). Fungi have also been identified in such contaminated environments and contribute to many biogeochemical transformations (Gadd and Fomina 2011; Passarini et al. 2022). It is well documented that fungi can interact with U, mainly by biomineralization and biosorption processes (Schaefer et al. 2021). In addition, fungi are good metal chelators forming metal–organic complexes through the secretion of low-molecular-weight carboxylic acids (oxalic, succinic, malic, and formic acids) (Gadd and Fomina 2011).

Different studies on in situ U bioreduction have been conducted at different mining sites to optimize the process and to study large-scale microbial reduction of U (Anderson et al. 2003; Istok et al. 2004). However, very few studies have reported the remediation of U contaminated sites at very low U concentrations (0.01–1 mg/L). Here, we describe the U-reduction potential of naturally occurring microbes in mine water from two former German U mines (Schlema-Alberoda and Pöhla, Wismut GmbH) for bioremediation in the concentration range between 1 and 0.01 mg/L. These low U concentrations resulted from controlled flooding-based remediation strategies applied to the mine water from these two U mines during Wismut remediation activities (Hiller and Schuppan 2008; Schuppan and Hiller 2012). According to the World Health Organization (WHO), the maximum admissible concentration of U in drinking water is limited to 0.03 mg/L (Frisbie et al. 2013; Ansoborlo et al. 2015; WHO 2022). This concentration may vary amongst European Union member states (Garboś and Świecicka 2015) and 0.5 mg/L when discharged into the aqueous environment (Wismut GmbH Umweltbericht 2021).

The main objective of the present study was to characterize the geochemistry, structure, and composition of the microbial community of mine water from the two former U mines in order to assess the U-bioremediation potential of the native microbial community. Exploring the links between the geochemistry and microbial diversity of U mine water will provide insights into how microbial communities survive and thrive in such extreme contaminated environments and help designing efficient bioremediation strategies. The second objective was to screen for optimal electron donors (glycerol, gluconic acid, and vanillic acid) to be used as biostimulators for the growth of U-reducing bacteria in the studied mine water. Gluconic acid and vanillic acid were identified in the studied mine water as wood-decay products (Baraniak et al. 2002). For comparison purpose, glycerol was used as reference electron donor previously described for its suitability for U removal by U reducing bacteria (Madden et al. 2007; Newsome et al. 2015; Coral et al. 2022).

## Materials and methods

### Site description

Uranium was discovered in the Ore Mountains, a mountain range on the border between Saxony (Germany) and Bohemia (Czech Republic). The Schlema-Alberoda mine was one of the most important Wismut mining sites. From 1946 to the beginning of 1991, about 80,000 t of U were extracted, left behind a sub-surface mine area at a depth of 1800 m and a volume of 35 million m^3^ (Meyer et al. 2008; Hiller and Schuppan 2008; WISMUT GmbH Brochure 2015). Another important Wismut mining activity site located in this area was the Pöhla mine with a depth of 600 m below the surface and a volume of 1.5 million m^3^. This uranium deposit was only partially mined and produced around 1200 t of U from 1967 to 1990 (Schuppan and Hiller 2012).

After the cessation of active mining, flooding by inflowing infiltration water has been carried out and controlled in the Wismut mine of Schlema-Alberoda and Pöhla since 1991.

### Mine water sampling description

Fresh mine water samples were collected from two flooded subsurface mine shafts: (1) Schlema-Alberoda (50°37′32.5″N, 12°40′52.4″E) and (2) Pöhla (50°29′34.8″N, 12°49′07.1″E) in August and September 2020, respectively, by using the water, which is pumped to the surface. Since the major part of the flooding of the mines were completed in 1995 (Pöhla) and 2008 (Schlema-Alberoda), strong changes in the flow velocity of the subsurface water bodies are no longer expected. A total of 13 L of mine water were sampled per mine in sterile autoclaved screw-capped bottles at an authorised and secure point at each mine. Sampling at Schlema-Alberoda was conducted through an external pipe where water was pumped from inside the mine to the outside. At Pöhla, sampling was carried out inside the mine, through a pipe connected to the mine. In both mines, samples were taken after properly purging the pipe, discarding several water volumes in order to eliminate the residual water to obtain representative samples. The samples were transported to the laboratory at 4 °C and stored in a refrigerator at the same temperature on arrival until further processing.

### Mine water chemistry characterization

Different physicochemical parameters of water from the two mines were determined to link the microbial diversity and the geochemistry of the studied water samples. Mine water temperature was measured in situ using a conventional thermometer. pH and redox potential (*E*_H_) were determined in situ using a pH meter 3110 (WTW, Germany) with a BlueLine 16 pH microelectrode (Schott Instruments, Germany) and a micro redox electrode with platinum ring (ORP electrode, Mettler-Toledo InLab, Spain).

For the determination of the geochemical parameters, aliquots of each mine water were centrifuged at 4020 × *g* for 15 min (Hettich EBA 21, Germany) prior to the analysis. A volume of 50 mL was acidified with nitric acid (HNO_3_) and used to measure the total concentration of cations (Na, K, Mg, Ca, Al, Si, P, Mn, Fe, As, Ba, Th, U) by inductively coupled plasma mass spectrometry (ICP-MS, ELAN 9000, PerkinElmer, Germany). Furthermore, an aliquot of 15 mL was taken to measure the total concentration of anions (NO_2_^−^, NO_3_^−^, PO_4_^3−^, SO_4_^2−^, Cl^−^) by high-performance ionic chromatography (HPIC, Dionex Integrion, Thermo Fisher Scientific, USA). Total inorganic/organic carbon (TIC/TOC), dissolved organic carbon (DOC), and nitrogen were also quantified (Multi N/C 2100S, Analytik Jena, Germany).

### Thermodynamic calculation of the U speciation of the mine water

The analytical data, which were obtained from the untreated Schlema-Alberoda and Pöhla mine water, were used to calculate the predominant fields of the possible U species present in the environmental conditions. The Pourbaix diagrams were calculated using the geochemical speciation code Geochemist’s Workbench, version 17.0.1/Act2. The thermodynamic database used was the ThermoChimie database Version 10.a (Giffaut et al. 2014; Grivé et al. 2015).

In addition, abiotic controls consisted of sterile (autoclaved) mine water samples from Schlema-Alberoda amended with 10 mM glycerol, vanillic, and gluconic acid to investigate whether these electron donors affect the mine water chemistry. After 128 days, the analytical data of the microcosms were used for thermodynamic speciation calculation using the analogue database, data from the literature (Vulpius et al. 2006 for vanillic acid; Zhang et al. 2009 and Sawyer 1964 for gluconic acid), and the geochemical speciation code in the Geochemist’s Workbench (version 17.0.1/Act2).

### Cryo-time-resolved laser fluorescence spectroscopy (cryo-TRLFS) studies of mine water

Aliquots of 2 mL in plastic single-use cuvettes (Rotilabo, Carl Roth, Germany) were immediately shock frozen with liquid nitrogen and stored at − 20 °C. These aliquots were used to determine soluble U(VI) species in the water from both U mines by cryo-TRLFS. Cryo-TRLFS is a non-destructive technique and it does not change the chemical composition of the mine water. All the measurements were carried out under cryogenic conditions. The luminescence of soluble U(IV) in cryogenic conditions was measured with a laser energy of 300 µJ, frequency quadruplication at 266 nm, pulse width of 5 – 8 ns, and a frequency of 10 Hz using a Nd:YAG pulsed laser system (Continuum Inlite series, Continuum, USA). The luminescence spectra were detected using an iHR550 spectrograph and an intensified CCD-camera system (HORIBA Jobin Yvon, Edison, USA) in a wavelength range from 350 to 650 nm. The intrinsic luminescence properties of U(VI) are of great advantage for label-free U(VI) speciation studies. The disadvantage is quenching, caused by ligands (e.g. Cl^−^ or CO_3_^2−^). In order to reduce this quenching, the measurements were performed at a low temperature (− 120°C) (Steudtner et al. 2011)*.* Data were evaluated using the software OriginPro v9.7 2020 (OriginLab Corporation, USA). Collected spectra were analysed by parallel factor analysis (PARAFAC) (Andersson and Bro 2000; Drobot et al. 2015).

### Molecular analysis of the microbial communities

#### DNA extraction and rRNA gene sequencing

From each mine, a total of 13 L were collected in several sterile glass bottles and transported to the laboratory for all the analyses. For DNA analysis, 800 mL of mine water were filtered through sterile 0.45- and 0.20-μm pore size membrane (Membrane Filter, MF-Millipore®, Germany) filters. The filters were immediately frozen at − 20 °C. Three biological replicates per mine water sample were analysed. Each filter was cut into four pieces and each piece was aseptically placed in a 5 mL sterile screw-cap tube from the DNA extraction kit (DNeasy Power Water Kit, QIAGEN, Germany). DNA extraction was carried out according to the manufacturer’s protocol, with the temperature increase modification recommended by the manufacturer for obtaining fungal DNA. The DNA extraction was checked by agarose gel electrophoresis (0.75% w/v) and DNA concentration was determined using Qubit Fluorometer 4.0 (Thermo Fisher Scientific, USA) according to the manufacturer’s protocol. The samples were stored at − 20 °C until DNA amplification. After quality control, three out of the four DNA extractions from each filter were designated for further analysis. The DNA extracted from the filter pieces was pooled into a single 1.5-mL low-retention tube and considered as one biological replicate. The bacterial 16S rRNA gene was amplified using the forward primer 341F (5′-**CCTACGGGNGGCWGCAG**-3′) and the reverse primer 785R (5′-**GACTACHVGGGTATCTAATCC**-3′), targeting the hypervariable V3-V4 regions (Thijs et al. 2017). Fungal ITS gene amplification was performed using the forward primer ITS1F (5′-**CTTGGTCATTTAGAGGAAGTAA**-3′) and the reverse primer ITS2R (5′- **GCTGCGTTCTTCATCGATGC** -3′) (Op De Beeck et al. 2014).

PCR amplification, assembly, and sequencing of the libraries (Illumina Mi-Seq) were carried out in the STAB-VIDA laboratories (STAB-VIDA, Caparica, Portugal; https://www.stabvida.com/es).

#### Molecular data analysis

FastQC was used for quality control of the raw sequence data (Andrews 2010). 16S and ITS rRNA raw sequences obtained by Illumina MiSeq were analysed by QIIME2 v2020.8 (Quantitative Insights into Microbial Ecology) (Caporaso et al. 2010; Bolyen et al. 2019). DADA2 (Divisive Amplicon Denoising Algorithm 2) plugin was used to denoised the reads (trimming and truncating low quality regions; dereplicating the reads and filtering chimeras) (Callahan et al. 2016). Then, the reads were organized into Amplicon Sequence Variants (ASVs). Taxonomy was assigned based on a scikit-learn classifier pre-trained on SILVA (release 138 QIIME) for bacterial sequences (Quast et al. 2013) and UNITE (release 8.2) for fungal sequences (Nilsson et al. 2019) with a clustering threshold of 97% similarity. ASVs containing at least 10 sequence reads were considered as the dominant ASVs.

#### Statistical analysis

Alpha and beta diversity analyses were performed on MicrobiomeAnalyst (v4.1.3) (https://www.microbiomeanalyst.ca/ (accessed on 01 February 2022) (Dhariwal et al. 2017). To remove low quality and/or uninformative features that could be associated with sequencing errors or low-level contamination, a low count filter and a low variance filter were implemented on the data. ASVs with four read counts and representing 20% of the total counts were kept. The variance of read counts was assessed using the interquartile range, and any ASVs with a percentage of counts below the cutoff (> 10%) were excluded. In addition, the data were rarefied to the minimum library size. The microbiome was explored at the genus and phylum level but only the results at genus level are shown in this publication. Statistical results at phylum level can be found in the supplementary material. Chao1 and Shannon indexes were used to study alpha diversity. The statistical significance of the indexes was tested using the Kruskal–Wallis test. Beta diversity was also explored in a non-metric multidimensional scaling (NMDS) matrix and Permutational analysis of variance (PERMANOVA) by Bray–Curtis dissimilarities. PAST4 (v4.04) was used to perform principal component analysis (PCA) of the Hellinger-transformed data using the relative abundances of the taxonomic composition at phylum and genus level of the two samples (three biological replicates per sample), excluding taxa with a relative abundance below 1% (Harper 1999).

#### Data availability statement

Raw sequences used in this study are available in the Sequence Reads Archive (SRA) in the NCBI database under accession number PRJNA973613.

### Microbial uranium reduction: screening for suitable electron donors

To assess the potential of the indigenous microbial communities in Schlema-Alberoda mine water to remove soluble U(VI), biostimulation microcosm experiments of U reduction were setup. Glass serum bottles (1 L) were used for microcosms, filled with fresh Schlema-Alberoda mine water. Three different organic compounds were used singly as electron donors at 10 mM: glycerol (ROTIPURAN, Germany), vanillic acid or 4-hydroxy-3-methoxybenzoic acid (ACROS ORGANICS, USA), and gluconic acid sodium salt (ACROS ORGANICS, USA). These last two electron donors are particularly considered as typical wood-decaying products (Baraniak et al. 2002). The walls inside the mine are lined with wood, so the wood degradation products could be used as potential natural electron donors for microbial U reduction after controlled flooding of the mine. Glass serum bottles were degassed with nitrogen under sterile conditions and incubated (unshaken) at 28 (± 1) °C in the dark. To assess the key role of U reducing micro-organisms in this enzymatic process, controls were also considered. Untreated mine water was used as a control sample, as well as sterilised (autoclaved) mine water amended with electron donors. For the sampling, aliquots were taken carefully and slowly with a needle of a suitable length from the middle of the bottles to avoid touching the bottom and without disturbing the supernatant. This was done at the beginning and at the end of the experiment to measure changes in the total concentration of anions and cations. The aliquot was centrifuged at 4020 × *g* for 15 min with a centrifuge (Hettich EBA 21, Germany), which was inside the anaerobic chamber.

In addition, U, As, and SO_4_^2−^ concentrations were monitored over a period of 128 days, all by ICP-MS and HPIC. Furthermore, variations in *E*_H_ and pH parameters were monitored by the same methodology as mentioned in the “Mine water chemistry characterization” section.

## Results

### Geochemical characteristics of mine waters

The mine waters of Schlema-Alberoda and Pöhla were pH-circumneutral (6.6 and 7.3). The relatively low *E*_H_ (+ 139 mV and − 91 mV, respectively) indicate that reducing conditions existed in both samples. A high electrical conductivity (EC) was determined in the Schlema-Alberoda mine water (1.52 mS/cm) compared with the Pöhla sample (0.56 mS/cm), which is probably due to the higher concentration of dissolved ions, such as Na (99.5 mg/L and 31.4 mg/L), Mg (71.2 mg/L and 15.1 mg/L), and Ca (115 mg/L and 49.1 mg/L) (Table 1). Since the beginning of the monitoring of the Pöhla mine water by the Wismut GmbH, the U concentration decreased from 4.9 mg/L (Schuppan and Hiller 2012) to values around 0.01 mg/L. In contrast, the water from the Schlema-Alberoda mine still showed 1 mg/L U at the time of sampling. The As concentration has increased in both mines since the beginning of the flooding until our sampling from 0.1 mg/L (Schuppan and Hiller 2012) to approximately 1 mg/L. The determination of the anions showed relatively high SO_4_^2−^ values in the water of Schlema-Alberoda mine in contrast to the Pöhla water. The concentrations of NO_2_^−^, NO_3_^−^, and PO_4_^3−^ were low in both samples. The mine water from Pöhla showed a low Cl^−^ concentration (3.36 mg/L). Conversely, the value determined in the water from Schlema-Alberoda was 15 times higher at 56.1 mg/L. The determination of dissolved organic carbon (DOC) and total organic carbon (TOC) showed similarly low values for the water from both mines. The values for total inorganic carbon (TIC) (96.6 mg/L and 53.2 mg/L), on the other hand, were high in both the samples. In particular, TIC content in the mine water from the Schlema-Alberoda mine was two times higher than that from the Pöhla mine. The value for total nitrogen (TN) was below the detection limit in both mines.

**Table 1:** Chemistry of the Schlema-Alberoda and Pöhla mine water

	Schlema-Alberoda	Pöhla
Sampling date	11.08.2020	23.09.2020
pH	7.3 ± 0.1	6.6 ± 0.1
E_H_ [mV]	139 ± 20	− 91 ± 20
EC [mS/cm]	1.52	0.56
Temp. [°C]	24 ± 1	18 ± 1
Cations [mg/L]		
Na	99.5 ± 1.44	31.4 ± 0.26
Mg	71.2 ± 1.91	15.1 ± 0.6
Al	< 0.01	< 0.01
Si	7.76 ± 0.14	10.4 ± 0.97
K	10 ± 0.17	5.46 ± 0.08
Ca	115 ± 6.43	49.1 ± 1.68
Mn	1.44 ± 0.004	0.17 ± 0.002
Fe	0.99 ± 0.038	0.01 ± 0.0004
As	0.92 ± 0.017	1.15 ± 0.01
Ba	0.03 ± 0.0004	1.79 ± 0.015
Th	< 0.001	0.002 ± 0.0002
U	1.05 ± 0.05	0.01 ± 0.0001
Anions [mg/L]		
Cl^–^	56.1 ± 1.57	3.36 ± 0.1
NO_2_^–^	< 0.05	< 0.05
NO_3_^–^	< 0.05	0.075 ± 0.003
PO_4_^3–^	0.399 ± 0.01	0.1 ± 0.01
SO_4_^2–^	335 ± 2.68	0.5 ± 0.015
[mg/L]		
TIC	96.6 ± 3.53	53.2 ± 0.29
TOC	9.5 ± 0.03	4.8 ± 0.15
DOC	9.2 ± 0.04	4.9 ± 0.11
TN	< 0.1	< 0.5

*TIC* total inorganic carbon, *TOC* total organic carbon, *TN* total nitrogen, *E*_*H*_ redox potential, *EC* conductivity, standard deviation with *n* = 3

### Thermodynamic calculation

**Fig. 1:**
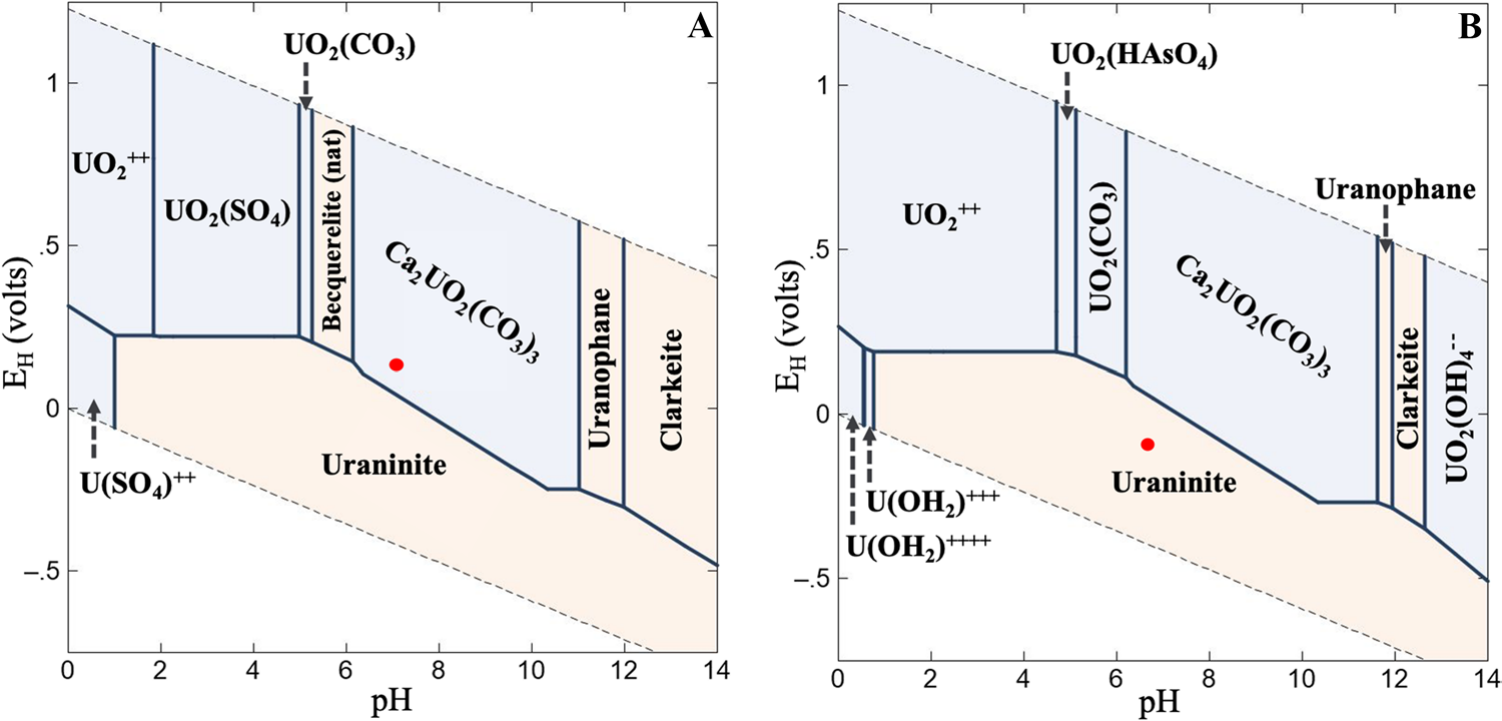
Pourbaix diagrams for the Schlema-Alberoda (**A**) and Pöhla mine water (**B**) after thermodynamic calculation using the geochemical speciation code Geochemist’s Workbench Version 17.0.1/Act2 and the analytical data. *E*_H_ and pH values of the mine waters were plotted into the diagrams (red points)

For the thermodynamic calculation of the predominance fields of uranium species, the analytical data of the Schlema-Alberoda and Pöhla mine water were used. The constructed corresponding Pourbaix diagrams (Fig. 1) were similar. Both mine water show an aqueous calcium uranyl carbonate species in the area characterized by a higher pH and a higher *E*_H_ limit, moving from + 130 mV at pH 6.2 to − 250 mV at pH 11 for the Schlema-Alberoda mine water and to − 270 mV at pH 11.6 for the Pöhla mine water. Due to the 100-fold lower U concentration in the Pöhla mine water, a precipitation of bequerellite was not predicted and the stability ranges of clarkeite and uranophane were smaller than Schlema-Alberoda mine water. The stability range of uraninite as a U(IV) mineral was, however, comparatively the same, since in both mine waters under reducing conditions the saturation limit of uraninite (Neck and Kim 2001) was exceeded, and mineral formation occurred. The plotting of the measured pH and *E*_H_ values into the calculated Pourbaix diagrams showed that Ca_2_UO_2_(CO_3_)_3_(aq) existed under the geochemical conditions in the Schlema-Alberoda mine water, whereas in the Pöhla mine water the formation of uraninite was predicted to occur due to the much lower *E*_H_ value.

### Determination of U species from cryo-TRLFS using PARAFAC analysis

**Fig. 2:**
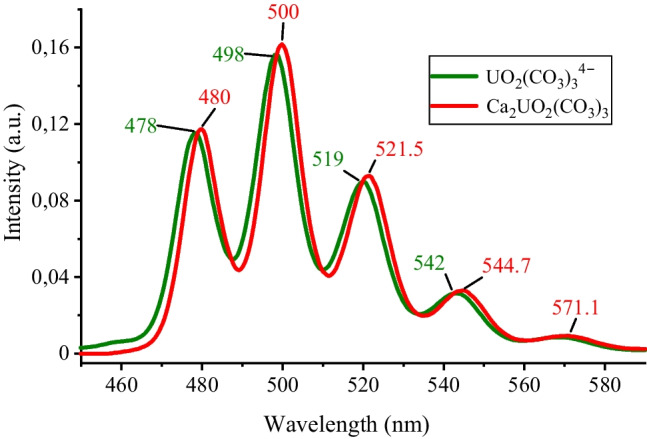
Deconvoluted spectra of the extracted aqueous species Ca_2_UO_2_(CO_3_)_3_ and UO_2_(CO_3_)_3_^4−^ of the Schlema-Alberoda mine water with determined emission wave lengths in the peak maxima

Previous U(VI) speciation modelling calculations represent an estimation of the real environmental conditions. Therefore, cryo-TRLFS was used to confirm the obtained U speciation. In the Pöhla mine water, no U signal was identified due to the low U concentration of 0.01 mg/L. However, in the Schlema-Alberoda mine water, the corresponding U(VI) spectrum was clearly identified (Fig. 2). To get an accurate insight into U(VI) speciation, the data were analysed using PARAFAC, providing information on the total number of U(VI) species present after deconvolution. The results provided two different U(VI) species. The first PARAFAC extracted species showed fluorescence bands at 479.5 nm, 500.0 nm, 521.5 nm, 544.7 nm, and 571.1 nm, matching with the fluorescence bands of the Ca_2_UO_2_(CO_3_)_3_(aq) species mentioned by Bernhard and co-authors (1998). The second species showed a slight shift of the emission bands to higher energies (lower wavelength). These bands at 478 nm, 498 nm, 519 nm, 542 nm, and 568 nm matched well with the uranyl carbonate complex UO_2_(CO_3_)_3_^4−^ (Wang et al. 2004).

### Microbial diversity analysis

#### Alpha and beta diversity

Species richness and diversity in the bacterial and fungal communities were examined (Table 1S) by analysing the average relative abundances of ASVs using the species richness estimate (Chao1) and Shannon (H´) diversity indexes for each sample. Moreover, beta-diversity analysis was performed through PERMANOVA and NMDS analysis, based on the Bray–Curtis index (Fig. 2SA, B).

Chao1 revealed a high richness in the bacterial community at genus level in both mine water, with no significant differences (*p* > 0.1) between them. In addition, a high bacterial diversity was observed by Shannon’s index, ranging from 2.540 to 2.699 in Schlema-Alberoda mine and 3.064 to 3.123 in Pöhla mine (Table 1S). The Shannon’s index showed a high fungal diversity in Pöhla mine water compared with Schlema-Alberoda mine water, the diversity values revealed a less diverse fungal community. However, the differences were still not significant (*p* > 0.1). For all the indexes, as expected, the values between replicates were similar.

Beta diversity revealed non-significant differences (*p* > 0.1) in bacterial (PERMANOVA, F: 114.17; *R*-squared: 0.96615; p: 0.1) and fungal (PERMANOVA, F: 21.232; *R*-squared: 0.84147; *p*: 0.1) community structure and abundance at genus level. Non-metric multidimensional scaling ordination (NMDS) based on Bray–Curtis dissimilarity matrices visualized the variation in bacterial (Stress: 0) and fungal (Stress: 9.211e-05) community composition between samples (Fig. 2SA, B). NMDS revealed no clear correlations between the compared microbial communities.

#### In situ bacterial community composition and structure

**Fig. 3:**
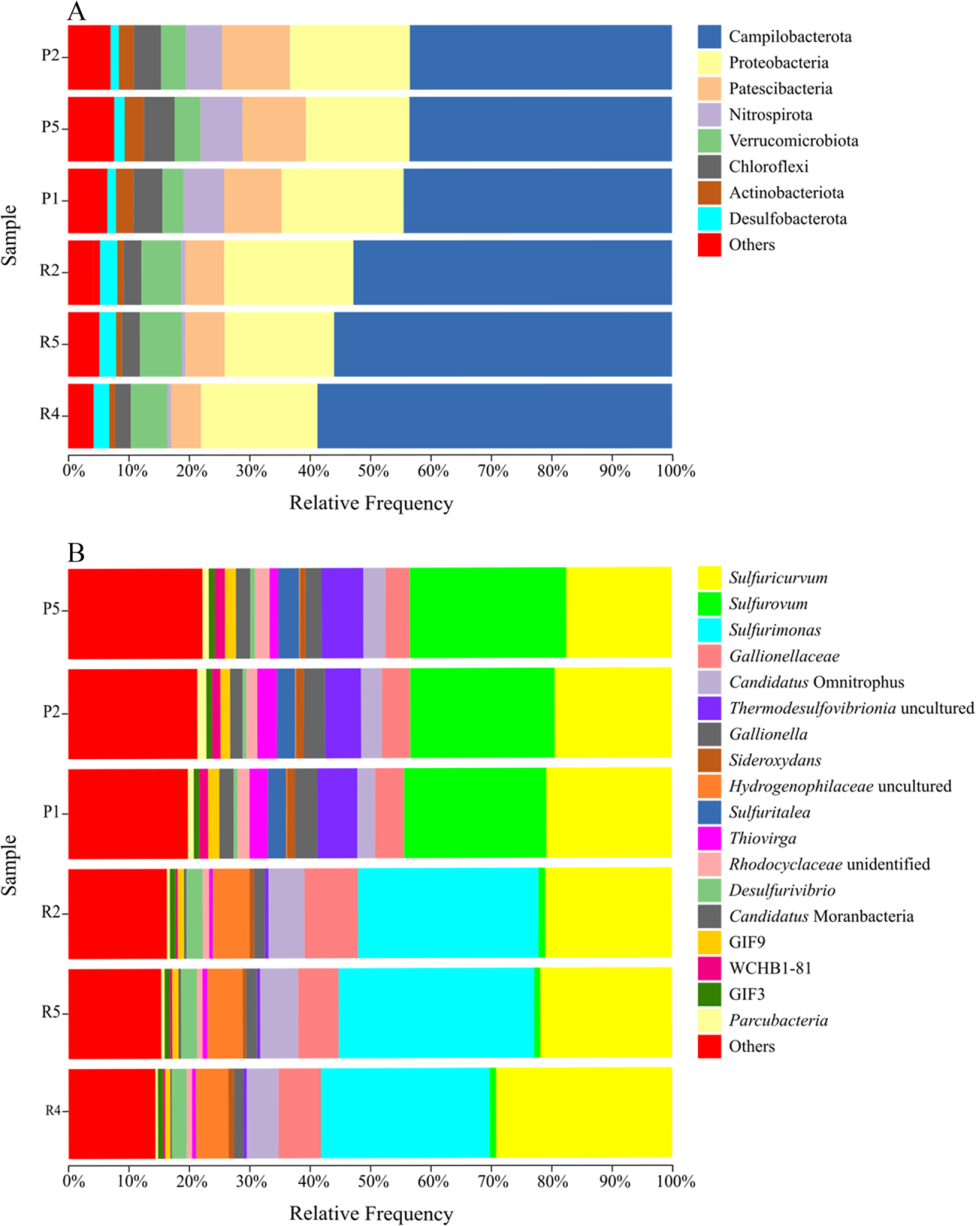
Barplot of the taxonomic distribution of bacterial diversity in the water samples from the Pöhla (P5; P2; P1) and Schlema-Alberoda mines (R2; R5; R4) at phylum (**A**) and genus (**B**) level. Each sample comprises three replicates. Only the phyla and genera identified in the three samples with > 1% relative abundance were included, while the remaining ones were included in “others”

A total of 1,825,744 raw sequences reads from the bacterial 16S rRNA gene were obtained after sequencing. An average of 268,112 raw sequences corresponded to the Schlema-Alberoda mine water and an average of 340,469 to the Pöhla water. Platform QIIME2™ (v2020.8) was used to analyse the sequences. Finally, 2611 ASVs were identified in total. Sequences were consistently affiliating to the following phyla for the water from both mines: Campilobacterota (49.11%), Proteobacteria (19.38%), Patescibacteria (8.60%), Verrucomicrobiota (5.04%), Nitrospirota (4.12%), Chloroflexi (4.01%), Actinobacteriota (2.11%), and Desulfobacterota (2.03%) (Fig. 3). Planctomycetota, Acidobacteriota, Bacteroidota, Acetothermia, and Firmicutes were also identified at a relative abundance of 1%.

A total of 377 different bacterial genera were identified. The Pöhla mine water was dominated by the genus *Sulfurovum* (24.94%), followed by *Sulfuricurvum* (19.57%), an uncultured genus of the class *Thermodesulfovibrionia* (6.65%), an unidentified genus of the family *Gallionellaceae* (4.56%), *Candidatus* Omnitrophus (3.39%), *Gallionella* (3.35%), *Sulfuritaela* (3.21%), *Thiovirga* (2.61%), *Candidatus* Moranbacteria (2.29%), an unidentified genus of the family *Rhodocyclaceae* (2.12%), GIF9 (1.80%), WCHB1-81 (1.56%), *Parcubacteria* (1.18%), and *Sideroxydans* (1.17%) (Fig. 3). Similarly, *Sulfuricurvum* (24.71%), an unidentified genus of the family *Gallionellaceae* (7.66%), *Candidatus* Omnitrophus (5.93%), *Gallionella* (1.74%), *Sulfurovum* (1.07%), amongst others with an occurrence of < 1%, were identified in the Schlema-Alberoda mine water (Fig. 3). However, the Schlema-Alberoda samples were was strongly dominated by the genus *Sulfurimonas* (30.68%). Uncultured genus of the family *Hydrogenophilaceae* (5.82%) and *Desulfurivibrio* (2.66%), were identified in the Schlema-Alberoda samples with a much lower occurrence compared to the Pöhla samples.

### In situ fungal community composition and structure

**Fig. 4:**
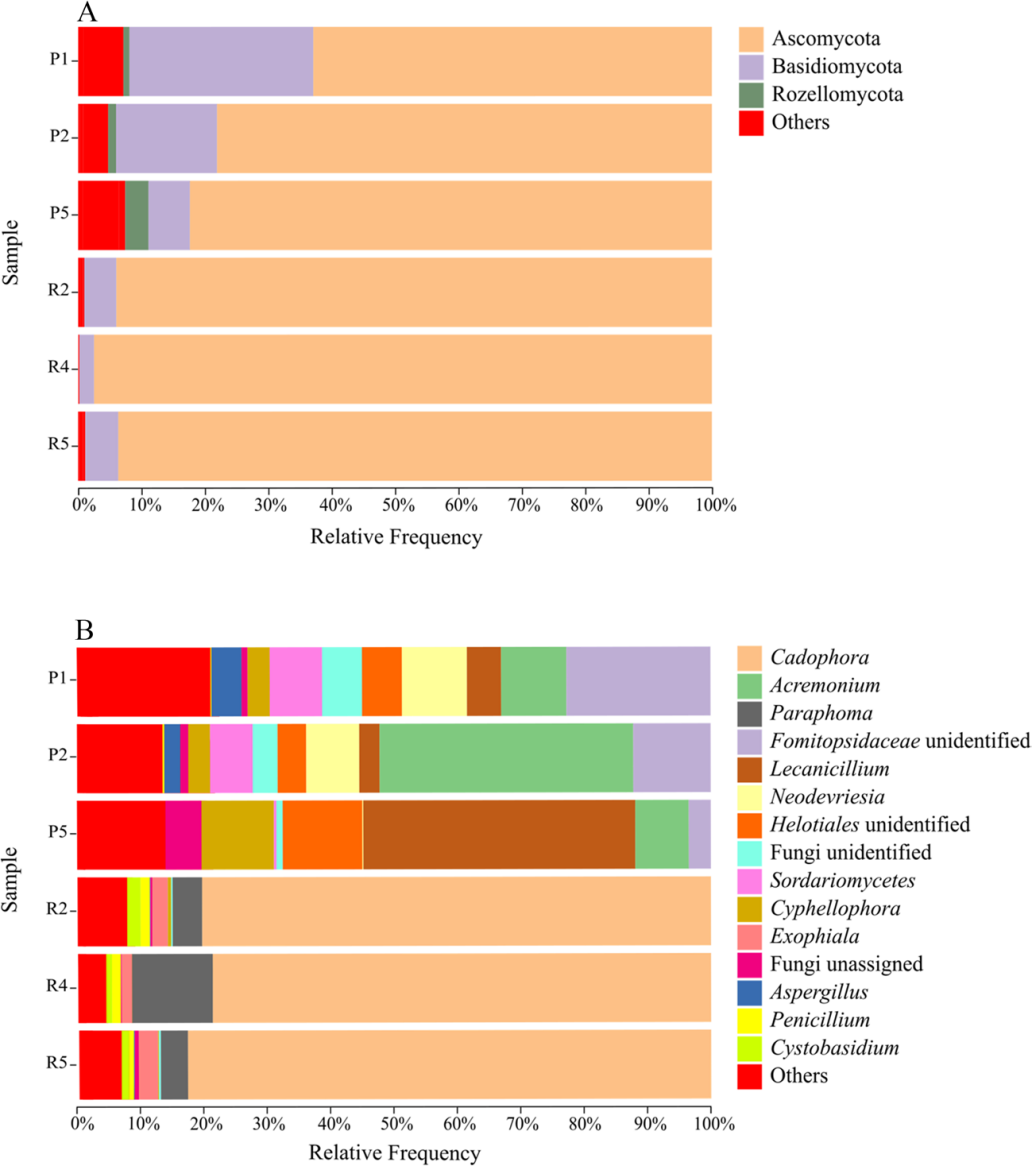
Taxonomic distribution of fungal diversity in the mine water from Pöhla (P1; P2; P5) and Schlema-Alberoda (R2; R4; R5) at phylum (**A**) and genus (**B**) level. Each sample comprises three replicates. Only phyla and genera identified in the three samples with > 1% relative abundance were included, while the remaining genera were included in “others”

The size of the fungal community was estimated by amplifying the ITS1 region of the rRNA gene, obtaining a total of 1,139,998 raw sequences reads. An average of 221,632 raw sequences corresponded to the Schlema-Alberoda mine water and an average of 158,366 to the Pöhla samples. A total of 373 ASVs were identified. At phylum level (Fig. 4), Ascomycota was strongly represented in the water from both mines with an average percentage of 79.75% in the Pöhla samples and 96.05% in the Schlema-Alberoda samples. In addition, Basidiomycota constituted 18.87% and 3.82%, respectively. The phylum Rozellomycota was mainly identified in the Pöhla water with an average occurrence of 1.30%.

In addition, a total of 119 different genera were identified in the mine water. The fungal community in the Pöhla mine water (Fig. 4) was characterized by *Acremonium* (29.66%), followed by an unidentified genus of the family *Fomitopsidaceae* (15.86%), *Lecanicillium* (14.42%), an unidentified genus of the order *Neodevriesia* (8.54%), *Helotiales* (7.94%), an unidentified genus of the class *Sordariomycetes* (6.91%), *Cyphellophora* (6.08%), an unidentified fungi genus (4.73%), *Aspergillus* (3.11%), and an unassigned fungi genus (2.49%). In contrast, the fungal community in Schlema-Alberoda (Fig. 4) was quite different compared to the Pöhla samples. The Schlema-Alberoda mine water was strongly dominated by the genus *Cadophora* (85.32%), followed by *Paraphoma* (8.80%), *Exophiala* (2.35%), *Cystobasidium* (1.37%), and *Penicillium* (1.30%).

#### Linking the microbial communities to the geochemical mine water parameters

PCA of the abundance matrices at the phylum and genus level (Fig. 3S and 4S, respectively) clearly showed a division between the major bacterial and fungal phyla in both mine waters. PCA at genus level showed the main influence of *Sulfurimonas* in Schlema-Alberoda and *Sulfurovum* in Pöhla. Interestingly, *Sulfuricurvum* influenced both mine waters. An unidentified genus of the family *Gallionellaceae* also showed a more pronounced effect on Schlema-Alberoda mine water. In addition, an uncultivable genus of the family *Thermodesulfovibrionia* had an influence on Pöhla mine water. In the PCA analysis, we did not observe major influences of specific fungal taxa on Schlema-Alberoda mine water, except for *Cadophora*, which had a slight impact. Conversely, in the Pöhla mine water, it was characterized primarily by the presence of *Acremonium*, followed by the influence of an unidentified genus of the family *Fomitopsidaceae*. In addition, we observed that bacterial and fungal groups plot together with the mines.

### Biostimulation of U-reducing bacteria: the effect of electron donors

In this study, it was observed that the water from both mines exhibited different U(VI) concentrations. The water from the Pöhla mine had a U(VI) concentration of 0.01 mg/L, that fits very closely to the allowed limit for drinking water in Germany (WHO 2022; Garboś and Świecicka 2015). However, the water from the Schlema-Alberoda mine presented higher U(VI) concentrations of about 1 mg/L, which should be decreased to the permitted levels. As a complement to the existing chemical treatment for the multiple mine water pollutants, the biostimulation of U(VI) reducing bacteria activity could lead to the removal of soluble U(VI) as U(IV) mineral phases within the mine. Therefore, preliminary U mine water bioreduction microcosms were designed and implemented using Schlema-Alberoda mine water (Table 1) amended with different electron donors (glycerol, vanillic acid and gluconic acid) in triplicates. The chemistry of the U mine water was determined at the beginning and at end of the experiment. In addition, a monitoring of the U(VI), As and SO_4_^2−^ concentrations, the *E*_H_ and pH values were carried out during the experiments (Table 2).

**Table 2:** Analysis of the most important cations and anions as well as the physico-chemical parameters of the original Schlema-Alberoda mine water during sampling and at the end of the microcosm experiment after the amendment of various electron donors

	SA	SAC	SA + G	ASA + G	SA + V	ASA + V	SA + K	ASA + K
pH	7.32	7.98	7.99	8.72	7.00	8.63	8.01	9.11
E_H_ [mV]	139	397	− 246	333	218	330	− 248	321
Cations [mg/L]								
Fe	0.99	0.60	0.05	0.11	0.32	1.19	0.35	2.47
As	0.92	0.97	0.46	0.65	0.40	1.13	0.17	0.58
U	1.1	1.15	0.01	0.70	0.10	1.25	1.37	0.62
Anions [mg/L]								
SO_4_^2−^	335	325	141	336	338	349	144	348

*SA* Schlema-Alberoda mine water during sampling, *SAC* Schlema mine water after 128 days, *SA* + *G* Schlema mine water + 10 mM glycerol, *ASA* + *G* autoclaved Schlema mine water + 10 mM glycerol, *SA* + *V* Schlema mine water + 10 mM vanillic acid, *ASA* + *V* autoclaved Schlema mine water + 10 mM vanillic acid, *SA* + *K* Schlema mine water + 10 mM gluconic acid, *ASA* + *K* autoclaved Schlema mine water + 10 mM gluconic acid,* E*_*H*_ Redox potential

Small changes in pH were observed, from circumneutral (pH 7.32) in the original mine water to slightly alkaline (pH 8) in the glycerol and gluconic acid amended microcosms. No difference in the pH of the sample amended with vanillic acid was noticed and it remained circumneutral. An increase in the pH of the control samples was also observed. Differences in *E*_H_ values compared to the initial conditions was detected in all the microcosms. At the end of the experiment, whilst the *E*_H_ of mine water microcosms amended with glycerol and gluconic acid reached very low values (− 246 ± 20 mV and − 248 ± 20 mV, respectively), the vanillic acid supplemented microcosm showed a higher *E*_H_ (+ 218 ± 20 mV) than the original mine water. However, the *E*_H_ of the controls increased considerably to + 397 ± 20 mV.

**Fig. 5:**
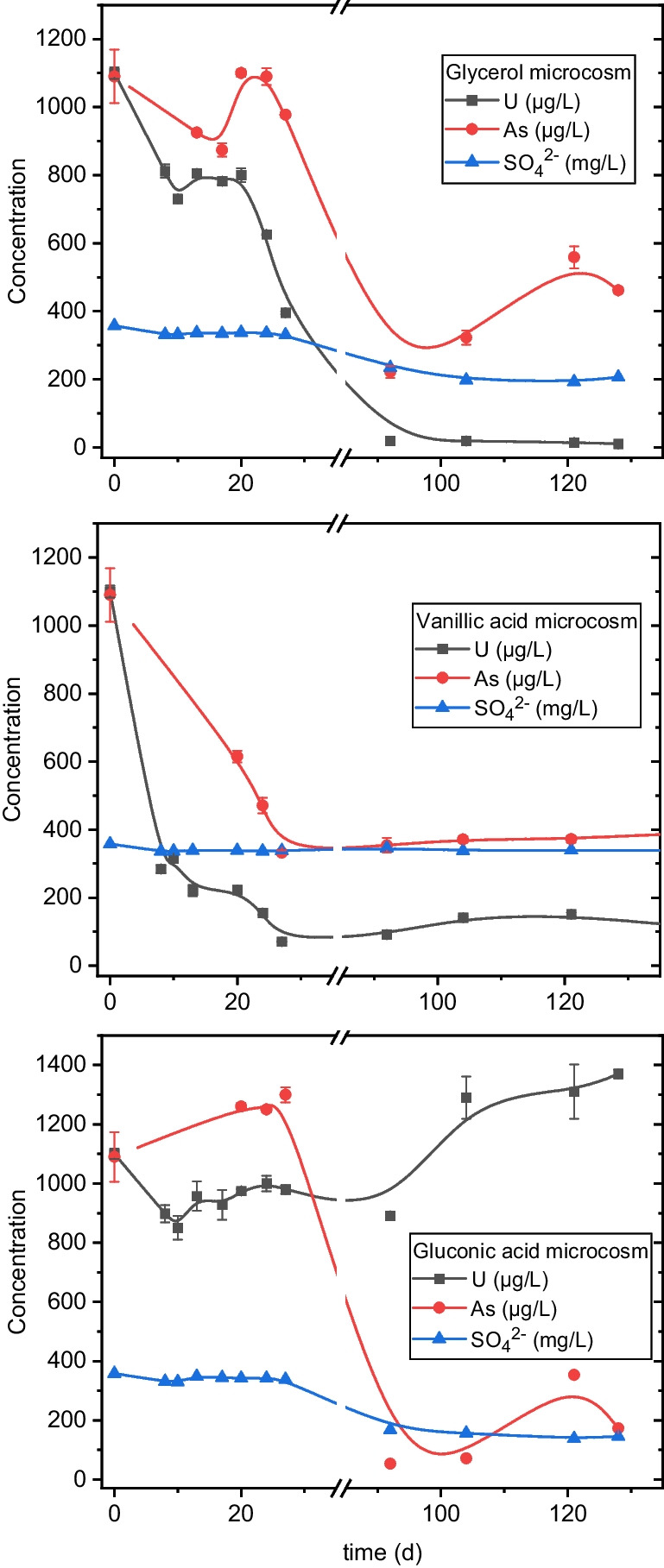
Changes in the kinetic of U, As, and SO_4_.^2−^ by biostimulation of the native community of micro-organisms after the addition of electron donors (glycerol, gluconic acid, and vanillic acid)

Figure 5 shows changes in the concentrations of U(VI), As and SO_4_^2−^ in the U mine water amended with the studied electron donors for 128 days. The concentration of dissolved U(VI) varies remarkably in the microcosms. The addition of glycerol to the microcosm led to a considerable decrease of the U(VI) concentration during the experiment. A reduction of 43% was already determined after 24 days. After 92 days, U(VI) was only detectable in small amounts, indicating a U(VI) reduction of 99%. Since it was not possible to collect samples between day 27 and day 92, it is unknown when exactly the U(VI) concentration decreased to 1%. In the microcosm supplemented with vanillic acid, the aqueous U concentration reduction was faster at the beginning of the experiment. Already on day 8, there was a considerable decrease of 76%. At the end of the experiment a notable U(VI) concentration decrease of 91% was also determined. In the gluconic acid microcosm, however, no U(VI) concentration decrease was observed.

The SO_4_^2−^ concentration decreased markedly after 27 days in the experiments with glycerol and gluconic acid. At the end of the experiment, a decrease of 58% and 57%, respectively, was determined. In contrast, using vanillic acid, no changes were observed. Both total Fe and As showed a notable decrease in their concentrations in each microcosm. The addition of glycerol to the mine water resulted in a decrease of about 95% of total Fe at the end of the experiment. In regard to As, the use of gluconic acid resulted in an 82% reduction of this oxyanion from the mine water, whereas when glycerol and vanillic acid were used, the removal rates of As were 50% and 43%, respectively. In brief, glycerol seems to show better results as an electron donor to stimulate the U-reducing bacterial community of the Schlema-Alberoda mine water than vanillic acid and gluconic acid.

The thermodynamic speciation calculation of abiotic controls of sterile (autoclaved) Schlema-Alberoda mine water amended with vanillic acid and gluconic acid showed that both electron donors do not affect the mine water chemistry under the given physicochemical conditions (Fig. 1S) since no complexation with U(VI) was predicted. U(VI) is neither coordinated to vanillic acid, nor to gluconic acid at the given pH of 8.63 and 9.11, respectively, at the end of the experiments. Strong calcium-uranyl-carbonate complexes such as Ca_2_UO_2_(CO_3_)_3_ and CaUO_2_(CO_3_)_3_^2−^ dominated the U speciation completely. Concerning glycerol, there were no thermodynamic data for complexation with U(VI). However, no formation of U(VI)-glycerol complexes were expected since glycerol has only three hydroxyl groups that do not deprotonate in aqueous solutions (Yu et al. 2021).

## Discussion

### Geochemical characteristics of the mine water

Studies on dammed or collected water from technical processes, such as the flooded mine water, often show a diverse and relevant microbial community, which could have a deep impact on the overall biogeochemical cycles of the elements in mine water and on its quality. In general, flooded mine water quality is determined by the solubility of the minerals from the mine and by chemical changes due to oxidation of the exposed ore and host rock (Bernhard et al. 1996). In addition, the methodology used during mineral extraction may also influence mine-water quality (e.g. acidification of host rock for ore extraction) (Arnold et al. 2011). Water from both mines (Schlema-Alberoda and Pöhla) showed a circumneutral pH. The Schlema-Alberoda water is characterized by a much higher SO_4_^2−^ concentration (335 mg/L) compared to that of the Pöhla sample (0.5 mg/L). The supply of SO_4_^2−^ is not limited due to the presence of sulphide mineralization. Nevertheless, we believe that the SO_4_^2−^quantity is insufficient to induce acidification within the system. The presence of multiple carbonates creates an excess of neutralization, which hinders the acidification of the water via the sulphide supply from the existing sulphide ores, as it was previously described by Hiller and Schuppan (2008). Ongoing studies are currently underway to investigate the reasons behind the higher SO_4_^2−^ concentration in Schlema-Alberoda compared to Pöhla mine water. The higher concentrations of Mg, Na, K, and Ca observed in the Schlema-Alberoda samples may originate from the chemical or microbiological alteration of granite (containing feldspar and plagioclase minerals) or dolomite (calcium magnesium carbonate) as described by Naumov et al. (2017) in the Schlema-Alberoda mine. Differences in *E*_H_ values may be due to the architecture of the Pöhla mine, being probably more “hermetic” than the Schlema-Alberoda mine, where infiltration of rainwater and O_2_ are possible. The considerably high As concentrations in both mines are probably caused by oxidation of the arsenic minerals in the ore veins, according to Paul et al. (2013).

The chemical behaviour of the uranyl ion in natural waters may be partly influenced by pH, *E*_H_, and dissolved ions (Bernhard et al. 1998). Thermodynamic calculations predicted a calcium uranyl carbonate complex [Ca_2_UO_2_(CO_3_)_3_(aq)] such as the dominant species in the Schlema-Alberoda mine water. By cryo-TRLFS measurements of the Schlema-Alberoda mine water combined with PARAFAC, two species were detected. We observed an analogy in the positions for Ca_2_UO_2_(CO_3_)_3_(aq) complexes. A slight shift of the fluorescence bands to the left for the second one matches with a uranyl carbonate complex [UO_2_(CO_3_)_3_^4−^] (Wang et al. 2004). Furthermore, our results are consistent with those of Bernhard et al. (1996, 1998, 2001), where calcium uranyl carbonate complex was reported as the major species in the Schlema-Alberoda water. Because of extremely low U concentrations in the Pöhla samples (0.01 mg/L), no detectable U signal was obtained by cryo-TRLFS measurements. On the other hand, by thermodynamic calculation of the predominance fields of U species, U is expected to form U(IV)-species, with uraninite as the end member.

### The impact of microbial populations on mine water biogeochemical processes

It is well known that the microbial community structure and function of U mine water are shaped by physicochemical factors such as pH, total organic carbon (TOC), dissolved oxygen (DO), concentrations of anions (e.g. NO_3_^−^, SO_4_^2−^), cations (e.g. Fe, Mn), and toxic heavy metals/metalloids (e.g. U, Pb, As). Schippers et al. (1995), Fields et al. (2005), and Shuaib et al. (2021) showed that heavy metal and radionuclide contamination reduce the microbial diversity of the environment. In this study, it was observed that the richness and diversity of the bacterial and fungal communities in the mine water from Schlema-Alberoda were lower compared to the mine water from Pöhla. These results align with those of prior cited studies, as the aqueous U(VI) concentration in Schlema-Alberoda is a hundred times higher than that in the Pöhla mine.

#### In situ bacterial community composition and structure

The bacterial community composition of water from both U mines displayed similarities at phylum level compared to that of other U-contaminated environments previously reported (Rastogi et al. 2010a, b; Zeng et al. in 2019; Lusa et al. 2019). However, the bacterial community of Schlema-Alberoda and Pöhla mine water exhibited a higher relative abundance of the following phyla: *Proteobacteria, Patescibacteria, Verrucomicrobiota*, and *Nitrospirota*. Interestingly, *Campylobacterota* was highly represented, with an average relative abundance of 49.11% in both mine waters (Fig. 3). The representative of these bacterial phyla has evolved mechanisms of resistance and tolerance to environmental toxicity of heavy metals and radionuclides. In addition, they play a major role in the biogeochemical cycles of elements such as S, N, and Fe, which subsequently affect U.

Bacteria involved in N/S redox cycling (nitrate reducers and sulphur oxidizers) from the phyla *Campilobacterota* and *Proteobacteria* were identified in water from both U mines. Abundant distribution of nitrate reducers and sulphur oxidizers from the genera *Sulfuricurvum*, *Sulfurovum*, and *Sulfurimonas* of the phylum *Campilobacterota* in water from both U mine has been observed. They were reported to be distributed in heavy metals and radionuclide impacted environments (Chang 2005; Shen et al. 2013; Zeng et al. 2019; Povedano-Priego et al. 2022) and to play a key role in the maintenance of reduced U species stability through coupling nitrate reduction to S-compound oxidation, and subsequently promote the growth of metal-reducing micro-organisms (e.g. Proteobacteria as SRB) (Chang 2005; Huang et al. 2021). Nitrate might negatively influence the microbial reduction of U(VI). Nitrate, ferric ion, and sulphate serve as thermodynamically more favourable final electron acceptors than U, and subsequently they would be reduced earlier than this radionuclide (Finneran et al. 2002). Therefore, anaerobic micro-organisms usually prefer nitrate as the first electron acceptor, followed by ferric iron and sulphate (Jroundi et al. 2020). In our study, the concentration of nitrate and nitrite remained below 0.07 mg/L, suggesting an adequate correlation of the microbial activity of these nitrate/nitrite reducers with the biogeochemical cycle of nitrogen. As pointed out in the PCA analysis, *Sulfuricurvum*, *Sulfurovum*, and *Sulfurimonas* strongly influence both mine waters, playing an important role (Fig. 4S).

Proteobacterial nitrate reducers and sulphur oxidizers including *Sulfuritalea*, *Thiovirga*, and an unidentified genus of the family *Hydrogenophilaceae* also constitute a considerable proportion in water from both U mines. They are well known for surviving in oligotrophic environments, and previously reported for their ability to reduce and tolerate metals (You et al. 2021; Bärenstrauch et al. 2022). *Sulfuritalea* can reduce nitrate to molecular nitrogen under anoxic conditions and oxidize thiosulphate, elemental sulphur, and hydrogen (Kojima and Fukui 2011). Furthermore, *Thiovirga* is a sulphur oxidizer (Ito et al. 2005). Peng et al. (2020) reported the role of the *Hydrogenophilaceae* family as beneficial and important in the sulphur cycle. *Hydrogenophilaceae* is able to oxidize sulphide compounds (e.g. S^2−^, HS^−^, and H_2_S) to SO_4_^2−^, which could be used by SRB (Peng et al. 2020). Its role in the reduction of nitrate to nitrite in microaerophilic members has also been reported (Orlygsson and Kristjansson 2014). Highly increased sulphate concentrations were observed in the Schlema-Alberoda mine water compared to the Pöhla mine water. The high sulphate concentration could be correlated with the role of sulphur oxidizing bacteria (SOB). The increased SO_4_^2−^ concentration could support the proliferation of SRB. For example, the phylum *Desulfobacterota* which contains several anaerobic genera of SRB including the genus *Desulfurivibrio. Desulfurivibrio* was mainly identified in the Schlema-Alberoda mine water where the sulphate concentration was higher (Jroundi et al. 2020). This is consistent with the assumption that SRB proliferated in the presence of higher sulphate concentrations. Moreover, an unidentified genus of the *Thermodesulfovibrionia* family (Nitrospirota) which couples H_2_ oxidation to sulphate reduction was also identified in the water samples (Rempfert et al. 2017; Nothaft et al. 2021; Umezawa et al. 2021). The reduced products of sulphate as hydrogen sulphides are able to chemically reduce U(VI) as the Fe-reducing bacteria (FeRB) do (North et al. 2004).

In addition to Fe oxidizing bacteria, U mine water also harbour an unidentified genus of the family *Rhodocyclaceae* which include FeRB with the ability to reduce U(VI) (Cummings et al. 1999; Porsch et al. 2009). Fe(III) reduction products have been reported to be able to chemically reduce U(VI) as well (Lovley et al. 1993; North et al. 2004; Wilkins et al. 2006). The abundant distribution of FeOB and FeRB in Schlema-Alberoda is correlated with its high Fe concentration. Furthermore, members of the *Rhodocyclaceae* family were reported to be able to grow lithotrophically by respiring U(VI) together with H_2_ oxidation and to be responsible for U(VI) bioreduction coupled with organic electron donors (Zhou et al. 2014).

Furthermore, alongside the phyla involved in the biogeochemical cycle of S, Fe, N and U, microbial diversity analysis has unveiled the presence of bacterial communities described for their adaptation to extreme environments including U-contaminated sites. Amongst them, the phylum reported as *Patescibacteria* has an ultra-small cell size, highly simplified membrane structures, and a greatly reduced metabolism highly adapted to U-contaminated environments by so far unknown mechanisms (Tian et al. 2020; Povedano-Priego et al. 2022). Nayak et al. 2021 identified sequences of unclassified *Candidatus* Moranbacteria (Parcubacteria), in radon- and heavy metal-contaminated water. *Candidatus* Omnitrophus (Verrucomicrobiota) is a chemolithoautotrophic bacterial genus that thrives in anoxic environments. This genus and its phylum have been previously reported by other authors in different contaminated environments (Underwood et al. 2022). The role they play is unknown, but they have generally been associated with environments contaminated by low concentrations of U, and could become a possible indicator for monitoring these contaminations as reported by Mumtaz et al. 2018. 16S rRNA gene analysis is valuable for detecting microorganisms in an environment and can be useful in designing efficient remediation technologies. However, for a better understanding of the microbial role in biogeochemical processes, future metagenomics and/or metatranscriptomics studies are suitable for this purpose.

#### In situ fungal community composition and structure

The fungal diversity of water from both U mines was dominated by Ascomycota (phylum with the highest number of fungal genera) and Basidiomycota. Rozellomycota was mainly identified in the Pöhla water mass but had a low relative abundance. These results are in agreement with those reported by Zirnstein et al. (2012) and Harpke et al. (2022) where these phyla were described in environments contaminated by U. Furthermore, Ascomycota, Rozellomycota, and Basidiomycota, have been reported in previous studies as phyla that could potentially play a key role in the decomposition and degradation of lignocellulosic biomass (Young et al. 2018; Liu et al. 2022). At the genus level*,* the water samples from the two U mines were characterized by the distribution of genera that have been previously reported in heavy metal and radionuclide-contaminated habitats. These include *Cadophora*, *Lecanicillium*, *Exophiala*, and unidentified genera of different taxa (e.g. order *Helotiales* (Ascomycota) and *Cystobasidium* (Basidiomycota), class *Sordariomycetes* (Ascomycota), and family *Fomitopsidaceae* (Basidiomycota) (Dos Santos Utmazian et al. 2007; Dirginčiute-Volodkiene and Pečiulyte 2011; Jasrotia et al. 2014; Văcar et al. 2021; Passarini et al. 2022). *Fomitopsis annosa* (*Fomitopsidaceae*) was reported to accumulate U (Nakajima and Sakaguchi 1993). To the best of our knowledge, this is the first study to describe the identification of genera such as *Neodevriesia* and *Cyphellophora* (Ascomycota) in these types of extreme environments. They are able to produce melanin, a substance that protects the cell and participates in the immobilization of metals and radionuclides such as U (Fogarty and Tobin 1996; Turick et al. 2008; Oh et al. 2021). The most representative genera based on their role in the removal and biomineralization of U phosphates were *Penicillium* and *Aspergillus* (Ascomycota) (Schaefer et al. 2021; Zhang et al. 2022). However, despite its high adaptive potential, *Penicillium* was poorly reported in the Schlema-Alberoda samples and *Aspergillus* in the Pöhla samples.

### Changes in mine water geochemistry by microbial biostimulation

The inner walls of Schlema-Alberoda mine are covered with spruce and pine boards to prevent the collapse of the floors. Although no data are available on the type of wood in Pöhla mine, an abundance of conifers left by mining activities can be assumed for both mines. During mining, wood degradation through microbial activity (mainly fungi) was observed. With beginning of the flooding process, the mine water comes into contact with the wood, causing the further degradation processes. The natural and fungal-mediated decomposition of the wood releases cellulose and lignin as well as low molecular weight molecules (carbohydrates, saccharic acids, vanillin, vanillic acid, and gluconic acid, amongst others) that may act as electron donors for U-reducing bacteria (Ander et al. 1980; Hedges et al. 1988; Baraniak et al. 2002; Mansour et al. 2020). Glycerol has previously been reported by other authors as an electron donor, in addition to lactate, acetate, methanol, and others (Madden et al. 2007; Newsome et al. 2015). These electron donors might stimulate the growth of SRB of the phylum Desulfobacterota (e.g. *Desulfurivibrio)*, distributed in minor proportions in the Schlema-Alberoda mine, that may play an important role in U(VI) reduction (Chang et al. 2001; Geissler and Selenska-Pobell 2005; Moon et al. 2010). Biostimulation is a simple and effective bioremediation strategy that has previously been reported in situ and at laboratory scale by other authors (Yabusaki et al. 2007; Williams et al. 2013; Xu et al. 2017). In order to study the potential of the natural microbial community in the reduction of U(VI) in Schlema-Alberoda mine water, we amended a set of anoxic mine water microcosms with three different electron donors (glycerol, gluconic acid and vanillic acid).

In terms of redox potential, the glycerol and gluconic acid amended microcosms became reduced reaching strong negative *E*_H_ values (− 246/ − 248 mV) at the end of the experiment. These values are broadly in line with the redox couple sulphate reduction and are supported by the removal rate of sulphates of about 58% for both microcosms. In the case of glycerol system, the U and Fe removal ratio was of about 90 and 95%, respectively, indicating the efficiency of this electron donor as stimulant of microbial reduction of these elements as it was described in different works (Newsome et al. 2015). Nonetheless, no U removal was detected in the gluconic system, which could be explained by the fact that this electron donor is not suitable for microbial U reduction. Nevertheless, in the case of vanillic acid amended microcosm the positive *E*_H_ value (218 mV) does not promote sulphate reduction as no decrease on the concentration of this anion was observed at the end of the experiment. However, a decrease of Fe, As and U concentrations was observed. The Fe reduction could be due to microbial activity as the Fe redox couple is on line with the *E*_H_ value of the studied system. The Fe reduction leads to the formation biogenic Fe oxides, which would remove U and As by co-precipitation and/or adsorption.

At the end of the experiment, remarkable changes were observed in the microcosm doped with glycerol where U(VI) concentration was reduced by ~ 99%. The concentration of total Fe (~ 95% reduction), SO_4_^2−^ (~ 58% reduction) and *E*_H_ were affected as well, mainly by glycerol. It suggests that biostimulation with glycerol could promote the growth of SRB and FeRB which may be involved in U(VI) reduction. Madden et al. (2007) and Newsome et al. (2015) reported similar U reduction rates using glycerol phosphate and glycerol. Glycerol seems to be the most efficient electron donor for the stimulation of bacterial populations with potential in the U removal and the bioremediation in Schlema-Alberoda mine water.

## Conclusions

To sum up, our study aimed to characterize the geochemistry and the native microbial community of the water from two former U mines. In addition, we carried out a screening test for electron donors to be used for the design of future U bioremediation strategy based on the biostimulation of U-reducing bacteria. Microbial diversity analysis revealed the distribution of bacterial populations with a key role in the biogeochemical cycles of relevant element for U reduction (e.g. sulphate reducers, iron reducers, iron oxidizers, nitrate reducers, and metal reducers). Thus, our results show that Fe and U, as well as SO_4_^2−^, could influence the differential diversity of the microbial community of the waters from the two mines as they are correlated with the biogeochemical cycles of these elements. In addition, mine water harbours wood degrading fungal communities providing potential electron donors, which promote the growth of U reducing bacteria. The elucidation of the overall bacterial diversity and the chemistry of the water from these mines could help the correct design of U bioremediation strategies.

The bioreduction of U(VI) in glycerol amended water from the Schlema-Alberoda mine based on the biostimulation of indigenous bacterial communities could be a viable alternative for U removal. We also observed that high levels of soluble U (99%), Fe (95%) and SO_4_^2−^ (58%) are removed by the use of glycerol as an electron donor. Glycerol probably stimulates the native micro-organism community by reducing soluble U(VI) to insoluble U(IV). Further ongoing studies will fully explore the U bioreduction processes through the microscopic and spectroscopic characterization of the reduced U solid phases and identification of the microbial communities actively involved in U removal.

### Supplementary Information

Below is the link to the electronic supplementary material.

Supplementary file1 (DOCX 756 kb)
