# Supplementary material for: Biostimulation of indigenous microbes for uranium bioremediation in former U mine water: multidisciplinary approach assessment
Source: Environ Sci Pollut Res Int. 2023 Dec 29;31(5):7227–45. doi: 10.1007/s11356-023-31530-4 (PMC10821841; doi:10.1007/s11356-023-31530-4)
Supplement: Supplementary file 1 — Supplementary file1 (DOCX 756 kb) [file 11356_2023_31530_MOESM1_ESM.docx]

# Supplementary

**Table 1S.** Alpha diversity index (Shannon) and richness (Chao1) of bacteria and fungi at genus level in water from the Schlema-Alberoda mine (R2; R4 and R5) and the Pöhla mine (P1; P2 and P5).

|  |  | Shannon | Chao1 |
| --- | --- | --- | --- |
|  |  |  |  |
|  | P1 | 3.064 | 220 |
|  | P2 | 3.091 | 217 |
|  | P5 | 3.123 | 224 |
| Bacteria |  |  |  |
|  | R2 | 2.699 | 187 |
|  | R4 | 2.540 | 182 |
|  | R5 | 2.589 | 169 |
|  |  |  |  |
|  | P1 | 2.666 | 42 |
|  | P2 | 2.192 | 42 |
|  | P5 | 1.871 | 30 |
| Fungi |  |  |  |
|  | R2 | 0.961 | 29 |
|  | R4 | 0.860 | 34 |
|  | R5 | 0.830 | 23 |

**Fig. 1S**: Thermodynamic speciation calculation of U(VI) with 10 mM vanillic acid and gluconic acid amended to sterile (autoclaved) Schlema-Alberoda mine water using the analogue database, data from the literature and the geochemical speciation code Geochemist's Workbench (version 17.0.1/Act2).

**
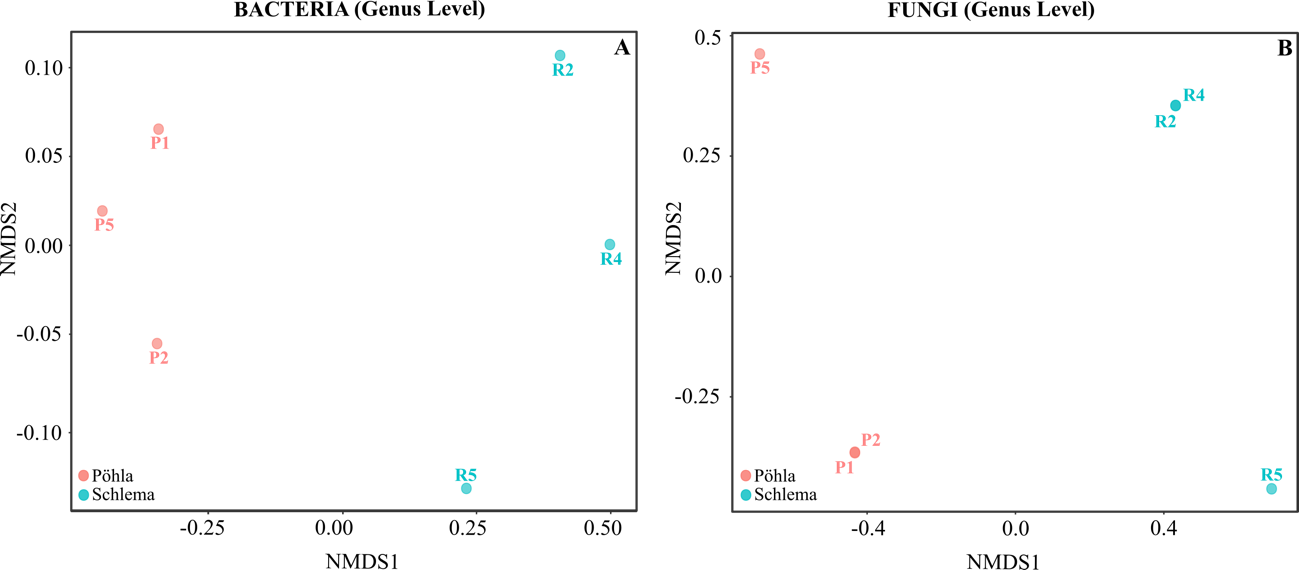
**

**Fig. 2S**. Bray–Curtis-based NMDS and PERMANOVA results of bacterial (A) and fungal (B) communities. Schlema-Alberoda (R2; R4; R5) and the Pöhla mine water (P1; P2; P5).

*
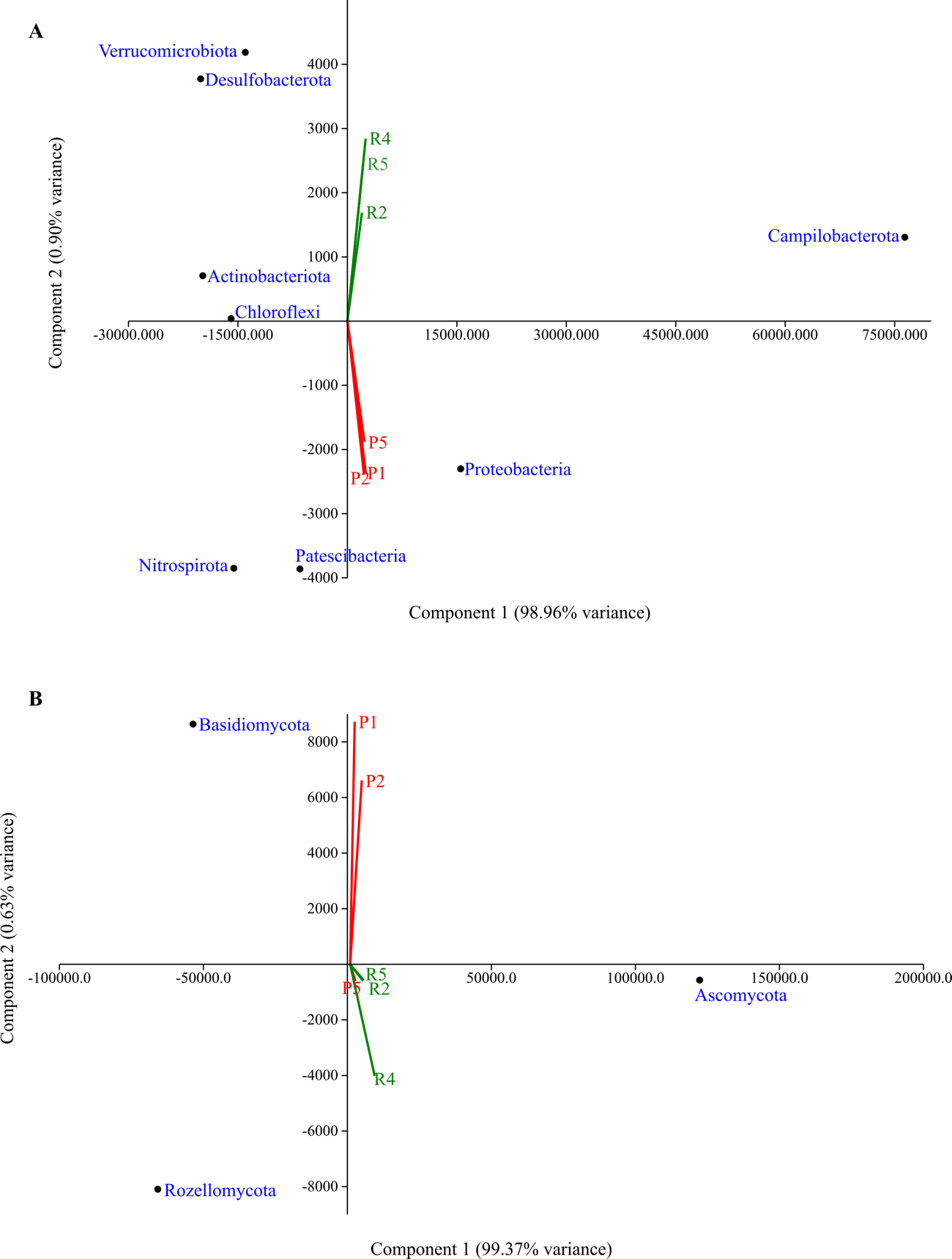
***Fig. 3S.** Principal component analysis (PCA) plot of bacterial (A) and fungal (B) communities at phylum level comparing the microbial community structure of the mine water from the Schlema-Alberoda (R2; R4; R5) and Pöhla mines (P1; P2; P5).

*
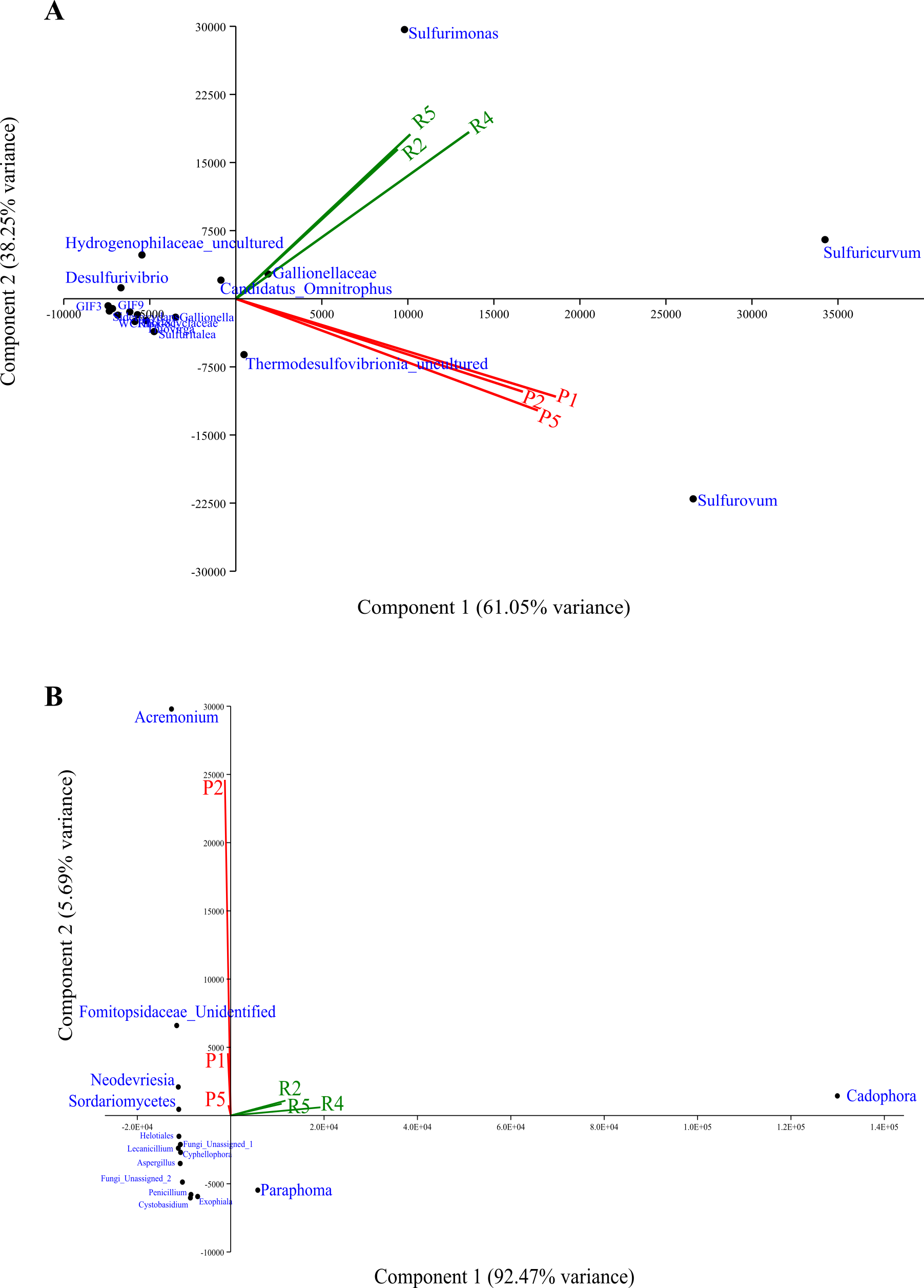
*

**Fig. 4S.** Principal component analysis (PCA) plot of bacterial (A) and fungal (B) communities at genus level comparing the microbial community structure of the mine water from the Schlema-Alberoda mine (R2; R4; R5) and the Pöhla mine (P1; P2; P5).
